# Bowman layer transplantation in the treatment of keratoconus

**DOI:** 10.1186/s40662-018-0117-y

**Published:** 2018-09-12

**Authors:** Diana C. Dragnea, Rénuka S. Birbal, Lisanne Ham, Isabel Dapena, Silke Oellerich, Korine van Dijk, Gerrit R. J. Melles

**Affiliations:** 1 Netherlands Institute for Innovative Ocular Surgery (NIIOS), Laan op Zuid 88, 3071AA Rotterdam, The Netherlands; 2 Melles Cornea Clinic, Rotterdam, The Netherlands; 3 Amnitrans EyeBank, Rotterdam, The Netherlands; 4 NIIOS-USA, San Diego, USA

**Keywords:** Cornea, Advanced keratoconus, Bowman layer, Penetrating keratoplasty, Deep anterior lamellar keratoplasty, Crosslinking, Intracorneal ring segments

## Abstract

Several treatment options corresponding to the grade of keratoconus have been established. These are ultra-violet corneal crosslinking and intracorneal ring segments for mild to moderate keratoconus, and penetrating keratoplasty or deep anterior lamellar keratoplasty for the more advanced cases of keratoconus.

Bowman layer transplantation was developed as a procedure for patients with advanced, progressive keratoconus. The technique consists of transplanting an isolated donor Bowman layer into a mid-stromal pocket of a keratoconic cornea resulting in corneal flattening and stabilization against further ectasia. Thus, it aims at corneal stabilization in eyes with advanced keratoconus, and enabling continued contact lens wear for normal visual functionality. By being a sutureless procedure and using an acellular graft, it potentially avoids commonly known suture and graft-related complications of penetrating or deep anterior lamellar keratoplasty.

The treatment seems to be a promising option in the management of advanced keratoconus in order to postpone or prevent a more invasive corneal surgery, while minimizing the risk of complications and allowing less stringent surveillance and less intensive medical therapy.

## Background

Several treatment options corresponding to the grade of keratoconus (KC) have been established [1]. These are ultra-violet corneal crosslinking (UVCXL) and intracorneal ring segments (ICRS) for mild to moderate KC, and penetrating keratoplasty (PK) or deep anterior lamellar keratoplasty (DALK) for the more advanced cases of KC [2–9].

UVCXL has been introduced in 2003 and its purpose is to freeze the evolution of the disease and postpone or prevent the need for corneal transplantation [2, 10, 11]. The results of the procedure have been encouraging, with approximately 80–90% of treated eyes reaching topographic stability [3], but the procedure may only be indicated in corneas with minimum corneal thickness of 400 μm. Although techniques are being developed to treat thinner corneas as well [4], it may be less suitable for more advanced KC, given that the rates of treatment failure and vision-threatening complications may increase [5].

The use of ICRS for KC was first reported in 2000 [6]. By reshaping the cornea, the segments may improve the corneal optics, while it may also confer some amount of support, possibly helping in postponing or avoiding corneal transplantation [7, 12]. Nevertheless, eyes with severe corneal thinning and steepening may be less eligible for the procedure [8].

For patients with advanced KC, PK and DALK are still currently used as the only options of treatment, in spite of well-known postoperative difficulties such as wound healing and suture related problems, tectonic instability, the risk of allograft rejection, a chronic steroid use that may predispose to cataract formation and glaucoma, and disappointing visual results [1, 9]. Advanced KC patients may, however, still have a subjectively acceptable contact lens (CTL)-corrected vision [13]. Consequently, they may still profit from reshaping the cornea and preventing further KC progression to enable continued contact lens wear with normal daily visual performance.

In KC corneas, changes in the organization of the stromal lamellae and unequal distribution of the collagen fibrillar mass, particularly around the apex of the cone, have been described [14]. Confocal microscopy studies have demonstrated a reduction in the number of keratocytes and stromal lamellae in KC compared to normal subjects, the reduction being greater in more advanced cases of KC [15]. Furthermore, the Bowman layer (BL) of these corneas consistently shows fragmentation, which are then filled with stromal collagen [16].

The physiologic purpose of the BL remains thus far somewhat unclear because numerous eyes have had their BL disrupted by laser refractive procedures without any significant consequences, and congenital absence of the BL has been reported in normal corneas [17]. On the other hand, it has also been suggested that the BL may be the strongest biomechanical element of the human cornea followed by the anterior third of the cornea [18]. As such, the BL may play a structural role in maintaining the shape/tectonic stability in KC corneas. Consequently, we hypothesized that a surgical approach in which the possible functionality of the BL in stabilizing the cornea could be restored, could potentially reinforce these thin and structurally fragile corneas. We developed a surgical technique in which an isolated BL graft is positioned inside a recipient KC cornea, sandwiched between the stromal layers above and below, while reshaping the anterior corneal surface in a flatter position [19]. The main treatment objective of the surgery, which was first described in 2014 and is called BL transplantation [19], is halting progression and reducing corneal steepness, which in turn, allows continued daily CTL wear (mainly scleral lenses) and preserve the present CTL-corrected vision, potentially postponing or avoiding the need for PK or DALK [20, 21]. By being a sutureless procedure and using an acellular graft, it potentially avoids all commonly known suture and graft-related complications of earlier techniques such as penetrating or deep anterior lamellar keratoplasty (PK and DALK, respectively) [1].

BL transplantation is indicated in eyes with progressive advanced keratoconus that are no longer eligible for UVCXL or ICRS given the corneal thickness or steepness. Potential candidates should have “acceptable” CTL-corrected vision and documented KC progression. What represents “acceptable” vision is likely to depend on the patient: in our experience, many patients would rather like to preserve the vision they have than undergo a PK/DALK with its risks and postoperative burden.

## Review

### Graft preparation

**Fig. 1:**
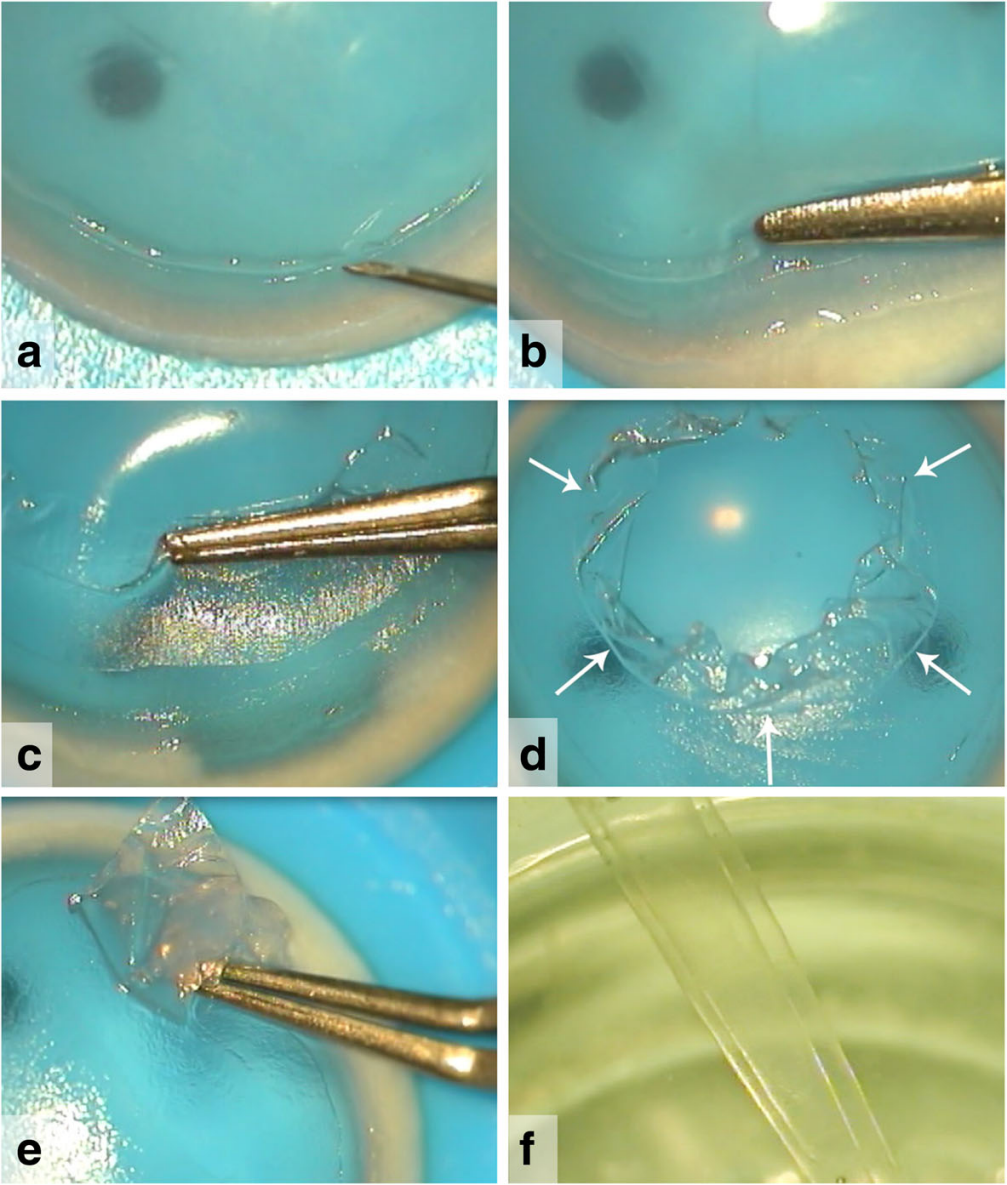
Surgical views of a Bowman layer (BL) graft preparation. A donor globe is mounted on a globe holder or a donor corneo-scleral rim is placed on an artificial anterior chamber with its epithelial side up. Corneal epithelial cells should be removed, after which, (**a**) just within the limbal area a superficial incision can be made over 360° with a 30G needle. (**b**) A peripheral donor BL edge is then lifted from the underlying anterior stroma using a single tip of a McPherson forceps. (**c-e**) Subsequently, by grasping the BL edge with the McPherson forceps via gentle slow movements in a circular manner, the entire BL is carefully peeled away to free the tissue from its underlying attachments. After preparation, the BL graft is evaluated and can be trephined if needed. (**f)** The BL graft tends roll into a single or double roll due to tissue elasticity

BL graft preparation was initially described in 2010 [22], and has remained largely unchanged since: BL grafts can either be prepared from whole donor globes (obtained less than 24 h postmortem, with corneas considered ineligible for PK) or from an anterior corneal button after stripping the Descemet membrane and endothelium for use in patients who were indicated for Descemet Membrane Endothelial Keratoplasty (DMEK) [23]. Donor globes or anterior corneal buttons (epithelial side up) are mounted on a globe holder or artificial anterior chamber, respectively, and the epithelium is removed using surgical spears (Fig. 1). Then, a 30-gauge needle is used to incise the BL, just within the limbal area, 360° around. The peripheral BL can be lifted and grasped with a McPherson forceps and then carefully peeled free from the underlying stroma, obtaining a 9- to 11-mm diameter BL graft (Fig. 1) [23]. Due to the inherent elasticity of the tissue, BL grafts tend to curl into a single or double roll with the epithelial border at the outside (Fig. 1). In the end, the donor BL graft is rinsed in 70% alcohol for 30 s and stored in organ-culture medium until the time of transplantation [23].

Recently, the use of a femtosecond laser for donor BL preparation has been evaluated. Preliminary results were encouraging, showing that the laser cut tissues demonstrated smoother / more regular edges compared to those prepared manually. However, the femtosecond laser prepared grafts were significantly thicker, containing some amounts of anterior stroma. The potential optical impact of these differences in graft morphology is presently unknown [24].

### Surgical technique

**Fig. 2:**
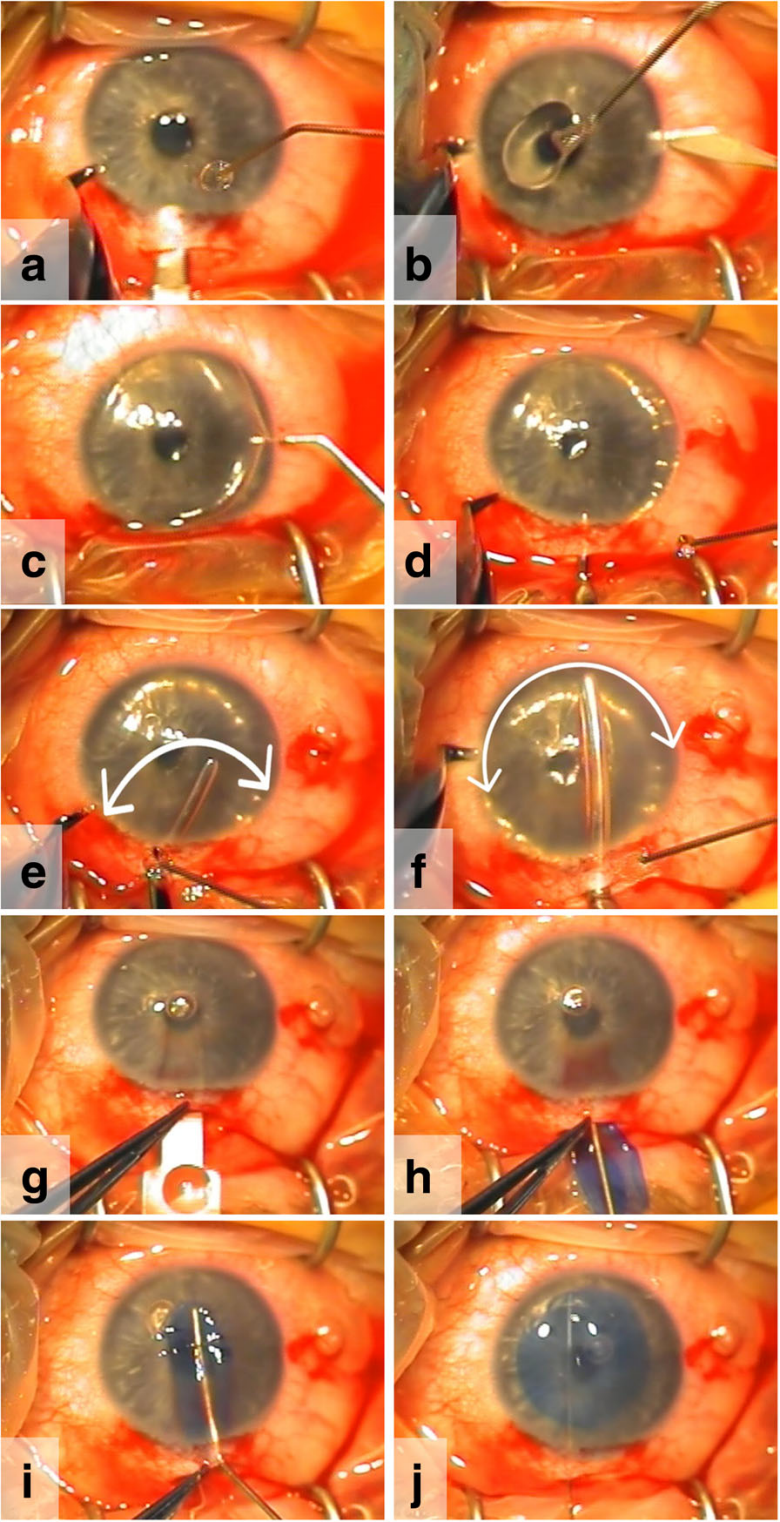
Intraoperative video-stills of a Bowman layer (BL) transplantation. (**a**) A scleral tunnel incision and (**b**) paracenteses are made. (**c**) Then, the anterior chamber is filled with air, and (**d**-**f**) a manually dissected mid-stromal pocket is created, using different sizes spatulas. (**f**) As an indication for the dissection depth, the ‘thin black line’ alongside the spatula can be used. After removing most air from the anterior chamber, (**g**, **h**) the BL graft is inserted into the pocket atop of a glide, and (**i**) then carefully unfolded and centered with an 30G cannula. (**j**) After complete unfolding and positioning, the BL graft is sandwiched between the anterior and posterior stromal layers. No sutures are necessary to fixate the graft or to close the tunnel incision

BL transplantations are performed under local anesthesia with the patient positioned in anti-Trendelenburg position, after an ocular massage and a Honan’s balloon for 10 min. The first step of the surgery is a superior conjunctival peritomy. Then 1–2 mm outside the limbus, a 5 mm partial thickness scleral tunnel is made and dissected up into the clear cornea using a crescent knife. Subsequently, a paracentesis is created and the anterior chamber is filled with air (Fig. 2) [19]. After this step, a manually dissected stromal pocket is created over 360° up to the limbus, using the technique as described for manual DALK [25]. For BL transplantation, a 50% dissection depth, which can be found using the “air-endothelial reflex” [25] is preferred (to minimize the risk of unintentional anterior or posterior perforation) (Fig. 2). Once this has been accomplished, most air is removed from the anterior chamber, and a surgical glide is threaded through the corneo-scleral incision into the dissected pocket. At the same time, the BL graft is again immersed in 70% ethanol for 30 s to remove all remnant cellular material, after which it is thoroughly rinsed with balanced salt solution (BSS), stained with Trypan blue, and placed atop of the glide, where it is pushed into the eye with the help of a cannula (Fig. 2). Once the graft is inside the stromal pocket, the glide is removed, and the graft is unfolded and positioned by manipulating it with the cannula, jets of BSS, and indentation on the corneal surface (Fig. 2). After a complete unfolding and positioning of the graft, the eye is pressurized with BSS, the conjunctiva is repositioned to the superior limbus and the eye is patched. No sutures are required.

Potential difficulties in the learning curve of the surgical technique are the midstromal manual dissection of these thin KC corneas, together with the graft handling. As the same manual dissection technique is used as with manual DALK, some experience with the manual DALK-surgery could be beneficial for the learning curve in performing a BL transplantation. Furthermore, familiarity with DMEK may aid in BL graft handling.

Postoperative medications include antibiotics for one week and a corticosteroid for the first month, after which the steroid may be tapered according to the surgeon’s discretion [19, 20].

### Clinical outcomes

**Fig. 3:**
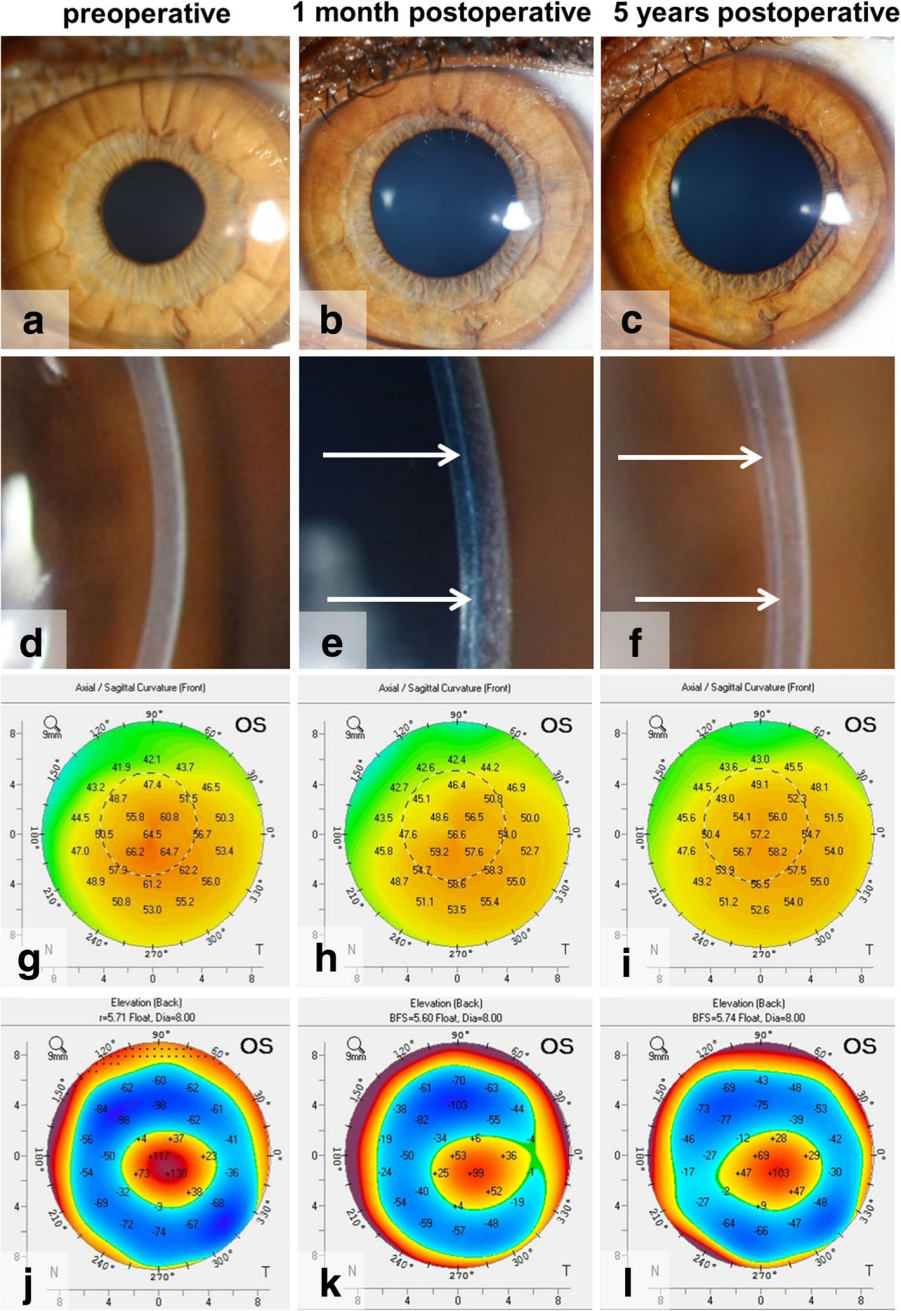
Pre- and postoperative clinical images of an eye that underwent BL transplantation. (**a**-**f**) Slit-lamp pictures, (**g**-**i**) anterior corneal topography and (**j**-**l**) posterior corneal elevation maps of an eye, (**a**, **d**, **g**, **j**) before and (**b**, **e**, **h**, **k**) at one month and (**c**, **f**, **i**, **l**) 5 years after Bowman layer (BL) transplantation. Slit-lamp imaging demonstrates (**a**) a clear cornea preoperatively, as well as postoperatively at (**b**) 1 month and (**c**) 5 years, while (**e**, **f**) the BL graft is visible as a thin white line within the host stroma (white arrows). (**g**-**i**) Corneal topography shows a flattening from (**g**) preoperatively to (**h**) the 1 month follow-up, and (**h**, **i**) stabilization thereafter. Likewise, (**j**-**l**) a decrease in posterior corneal elevation can be noticed from (**j**, **k**) before to 1 month postoperatively, after which (**k**, **l**) no changes occur up to 5 years after BL transplantation

BL transplantation is a relatively new KC treatment option with limited literature available. Single center clinical outcomes for BL transplantation are available up to 7 years after the surgery [21, 26]. In a first series of eyes in which BL transplantation was performed, operated eyes showed a significant flattening of the cornea of 8–9 D (on average) in maximum keratometry values in the first postoperative month, after which stabilization of the ectasia has been observed (Fig. 3) [20, 21]. Likewise, the posterior corneal curvature flattens and stabilizes thereafter (Fig. 3) [20]. Meanwhile, on slit-lamp examination the graft remains only vaguely visible as a thin white line (Fig. 3) [19–21].

Within a reported 5-year follow-up period, KC disease progression and/or severe complications could be avoided in 84% of the eyes [21]. The flattening of the cornea was accompanied by an improvement in spectacle-corrected visual acuity and a decrease in corneal higher order aberrations (especially spherical aberration) [27]. The mid-stromal positioning of the BL graft may however give some increase in corneal backscatter [27], which was found to occur up to 5 years after BL transplantation [21], and is possibly initiated by interface irregularities and/or differences in refractive indices between the BL graft and host stroma. Nevertheless, the clinical impact of this rise in corneal densitometry may be minimal given the objective and subjective lack of visual disturbance. In fact, CTL-corrected vision showed no changes from before to after BL transplantation [20, 21], and patients sometimes even experienced an improvement in their functional vision, since CTL wear became more comfortable as a result of the large amount of postoperative corneal flattening.

### Complications

So far, our experience with BL transplantation showed that the surgery may be a promising, minimally invasive approach to arrest KC progression; surgical manipulations are limited to the pocket within the recipient corneal stroma, no surface incisions are made, and no sutures are used to fixate the graft. Therefore, unlike PK or DALK, postoperative ocular surface complications or suture related problems do not occur. Furthermore, since the BL tissue is acellular, allograft reaction may be unlikely and topical steroids may be rapidly discontinued, minimizing the risk of glaucoma development or cataract formation.

The main intraoperative complication (the only encountered) may be a Descemet membrane perforation while dissecting the mid-stromal pocket, which is described to occur in 10% (2 out of a first series of 22 eyes) of the originally operated cohort of BL transplantation eyes [19, 20]. As with DALK, these perforations may be managed expectantly by aborting the operation, to allow healing, and re-attempting again at a later date. Alternatively, the surgeon may proceed with PK, depending on the size and position of the perforation [28].

Postoperatively, an unexpected complication is the occurrence of a corneal hydrops at 4.5, 6 and 6.5 years postoperatively in two patients (3 eyes), in a series of 20 eyes after successful BL transplantation. These patients had a history of severe eye rubbing and atopy and developed the hydrops despite no evidence of progressive steepening or thinning [26]. Therefore, also after BL transplantation, patients should be counseled about the possible impact of eye-rubbing, and allergies may need closer monitoring and treatment. No other postoperative complications have been observed [21, 26].

## Conclusion

BL transplantation aims at corneal stabilization in eyes with advanced KC, enabling continued CTL wear for normal visual functionality. The treatment seems like a promising option for the management of advanced KC in order to postpone or prevent a more invasive corneal surgery, while minimizing the risk of (long-term) complications and allowing less stringent surveillance and less intensive medical therapy.

## Abbreviations

BL: Bowman layer
BSS: Balanced salt solution
CTL: Contact lens
D: Diopters
DALK: Deep anterior lamellar keratoplasty
DMEK: Descemet membrane endothelial keratoplasty
ICRS: Intracorneal ring segments
KC: Keratoconus
PK: Penetrating keratoplasty
UVCXL: Ultra-violet corneal crosslinking
